# Impact of Dumpsites on the Quality of Soil and Groundwater in Satellite Towns of the Federal Capital Territory, Abuja, Nigeria

**DOI:** 10.5696/2156-9614-7.14.15

**Published:** 2017-06-22

**Authors:** Henry Olawale Sawyerr, Adedotun Timothy Adeolu, Abiodun Segun Afolabi, Oluwatoyosi Olalekan Salami, Biola Kazeem Badmos

**Affiliations:** 1 Department of Environmental Health Sciences, School of Allied Health and Environmental Sciences, College of Pure and Applied Sciences, Kwara State University, Malete, Nigeria; 2 Department of Environmental Management and Toxicology, School of Allied Health and Environmental Sciences, College of Pure and Applied Sciences, Kwara State University, Malete, Nigeria; 3 Centre for Ecological and Environmental Research Management and Studies, Kwara State University, Malete, Nigeria

**Keywords:** dumpsites, soil quality, groundwater quality, Federal Capital Territory, Abuja, Nigeria

## Abstract

**Background.:**

Urbanization, industrialization and changes in consumption patterns have compounded the problem of solid waste management in Nigeria. Poor waste management threatens the well-being and health of the local population, particularly those living adjacent to dumpsites.

**Objectives.:**

An assessment of the impact of dumpsites in a satellite town of the Federal Capital Territory, Abuja, Nigeria was carried out to determine the level of biophysical/chemical parameters (pH, temperature, conductivity, nutrients (calcium and magnesium), heavy metals (lead, chromium, zinc), and microbial burden) on the quality of soil and groundwater and their impact on health and the environment.

**Methods.:**

Soil and ground water samples were collected in four different dumpsites (Bwari, Gwagwalada, Kuje and Azhatta) with reference samples taken from the Federal Capital Territory, Abuja, and taken to the laboratory for biophysical/chemical analysis using standard methods.

**Results.:**

The results were compared with the national and World Health Organization (WHO) standard limits for soil and water respectively. Except for zinc, the average concentrations for heavy metals in the soil samples were higher in all four dumpsites than the permissible levels. Soil and water parameters that exceed the standard limits pose significant health and environment risks to nearby residents.

**Conclusions.:**

There is a need for raising the awareness of residents living close to dumpsites and those who use the well or nearby streams for domestic activities on the need to carry out adequate water treatment prior to its use.

## Introduction

Waste generation has been an issue for communities since the beginning of civilization. Waste is generated due to goods and service production and the utilization of natural resources.^1^ There are many barriers to the proper management of waste. In Nigeria, regular increases in population, industrialization and changes in consumption patterns have complicated solid waste management.^2^ The impact of poor waste management on human health and well-being cannot be overemphasized. Individuals living adjacent to dumpsites are at high risk due to the potential of waste to pollute water, food, land, vegetation and air.^3^ Waste comes from various sources: domestic residences, offices, institutions, commercial buildings, restaurants, agriculture, construction, and hospitals. The majority of the wastes generated from these sources ends up in dumpsites. Across many cities in Nigeria, collected wastes are usually burnt outdoors and ashes are poorly disposed of on-site. This act destroys the organic components and causes the oxidation of metals. The ashes left behind are enriched with metal, which results in pollution of the surrounding environment.^4^ The movement of contaminants from sites where wastes are disposed of to adjoining ecosystems is complex and involves biological and physico-chemical processes.^5^ Open dumpsites could be a source of microbial and toxic chemical pollution of the soils of the dumpsites. This can also pollute hand dug wells, posing serious health risks and leading to the destruction of biodiversity in the environment.^6^ Water can percolate through the refuse pile in the dumpsites. This leads to the formation of leachates that are enriched in nutrients (nitrogen, potassium and phosphorous), heavy metals, and other toxic substances, including cyanide and dissolved organics.^7^ The composition of the wastes influences the concentration of the leachates' constituents which may be adsorbed on to the soil during this diffusion.^8^ This process creates health hazards, soil and water pollution, and offensive odors, which increase with an increase in ambient temperature levels.^9^ The Federal Capital Territory of Nigeria (i.e. Abuja) is rapidly growing in terms of population and infrastructure. This rapid growth results in an increase in waste generation. Therefore, this study was designed to assess the significant impact of dumpsites in the selected areas in satellite towns of the Federal Capital Territory, Abuja, Nigeria in order to determine the levels of microbial and physicochemical parameters (pH, temperature, conductivity, nutrients (calcium and magnesium) heavy metals (lead, chromium, zinc, etc) on the quality of soil and ground water.

## Methods

### Study Area

**Figure 1:**
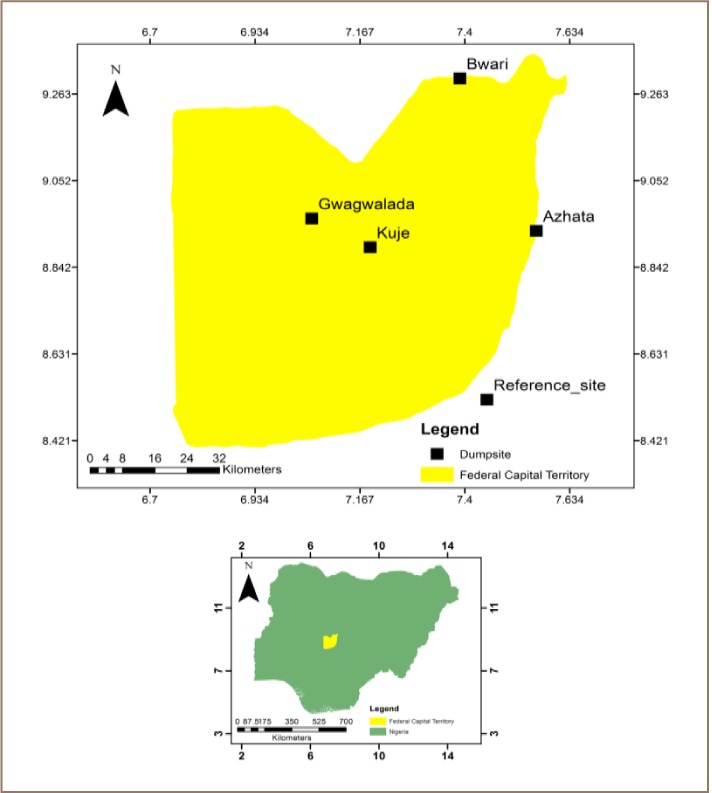
Map of Federal City Territory, Abuja Reference Site

The Federal Capital Territory, Abuja, Nigeria is located north of the confluence of the Niger River and Benue River. It lies between latitude 8.25 and 9.20 North and 6.45 and 7.39 East. It is bordered by Niger to the west and north, Kaduna to the northeast, Nasarawa to the east and south, and Kogi to the southwest. It has a landmass of approximately 7,315 km^2^ and is situated within the savannah region with moderate climatic conditions. It is currently composed of six local councils comprising the city of Abuja and five local government areas: Abaji, Abuja Municipal, Gwagwalada, Kuje, Bwari and Kwali. It has estimated population of 2,238,800. The anthropogenic activities which generate solid waste in the areas include construction, institutions, industries and households. The study was carried out in four different dumpsites: Gwagwalada (8.96 N, 7.06E); Bwari (9.30 N, 7.39 E), Azhata (8.93 N, 7.56 E) and Kuje (8.89 N, 7.19 E) which are located within the Federal Capital Territory satellite towns as shown in Figure 1. The reference site (8.52 N, 7.45 E) was selected outside the study area.

**Abbreviations**
*NESREA*: National Environmental Standards and Regulation Agency
*WHO*: World Health Organization

### Sample Collection and Analysis

The analytical phase involved the analysis of physicochemical, heavy metal concentrations and microbial parameters in soil and water samples collected from the dumpsites and the reference site to measure the impact of the dumpsite on the quality of the soil and water.

### Soil Samples

The soil samples were collected along transects lines which were drawn at each sampling site.^10^ Four samples of the top soil were taken along each of the transect lines. The samples were collected within 500 mm depth at the dumpsites and reference site and placed in a black polythene bag. The soil samples were then air dried, crushed, passed through a sieve 2 mm in diameter and put in clean polythene bags and stored at room temperature for laboratory analysis. The physicochemical qualities of the soil samples were determined according to the Association of Official Analytical Chemist's standard methods.^11^ The assessed parameters were pH, temperature, moisture content, electrical conductivity, chlorides, sulphides, nitrates, magnesium, potassium and calcium, ammonium. The heavy metals analysis was carried out using hydrochloric acid digestion. An atomic absorption spectrophotometer (model Philips PU 9100) with a hollow cathode lamp and a fuel rich flame (air acetylene) was used for the determination of metal ion concentrations. Each sample was aspirated and the mean signal response recorded at the metal ion's wavelength. The microbiological analysis of the soil samples was performed by the determination of the total heterotrophic bacteria, total coliform, Escherichia coli, Shigella and Salmonella according to the modified methods of Oyeleke and Manga and Rabah et al.^12^,^13^ The isolated bacteria were identified and characterized using Bergey's manual of classification.^14^

### Water Samples

Four water samples were collected from nearby wells about 20 m away from the selected dumpsites. A 50 ml representative sample was then collected in a plastic bottle.^15^,^16^ The procedure was repeated for the water sample taken at the reference site (serving as the control). Water samples were preserved in 5% v/v nitric acid on arrival at the laboratory, where they were analysed for the selected physical, chemical and biological properties. The physicochemical quality of the water samples (effluent from the dumpsites into the water source) was determined using the American Public Health Association standard methods.^17^ These parameters included pH, temperature, total dissolved solids, total suspended solids, electrical conductivity, hardness, dissolved oxygen, biological oxygen demand, chlorides, sulphates, nitrates, magnesium, potassium, calcium, and ammonium. The heavy metals analysis was carried out using hydrochloric acid digestion. An atomic absorption spectrophotometer (model Philips PU 9100) with a hollow cathode lamp and a fuel rich flame (air acetylene) was used for the determination of metal ion concentrations. Each sample was aspirated and the mean signal response recorded at the metal ion's wavelength. The microbiological analysis of the water samples was performed by the determination of total heterotrophic bacteria, total coliform, Escherichia coli, Shigella and Salmonella according to the modified methods of Oyeleke and Manga and Rabah et al.^12^,^13^ The isolated bacteria were identified and characterized using Bergey's manual of classification.^14^

All reagents were analytical grade (British Drug Houses, Poole, England). Glassware was washed with detergent, rinsed with distilled water, soaked in 10% nitric acid for 24 hours and rinsed. The atomic absorption spectrophotometer was calibrated using the calibration curve method in which standard solutions of at least three different concentrations were prepared to measure the absorbances of these standard solutions and to prepare a calibration curve from the obtained values. All data obtained were presented as descriptive statistics and compared with the national guideline limits set by the National Environmental Standards and Regulation Agency (NESREA) and the World Health Organization (WHO).

## Results

### Soil

The pH values of the soil samples from the selected dumpsites were similar, ranging from 7.1 to 7.8 (*Table 1*), similar to the values reported by Osakwe and Otuya.^18^ Conductivity values ranged from 81–137 μs/cm, but were higher when compared with the reference site (control). These values were in the range of values reported by Ovasogie and Omoruyi.^19^

**Table 1:**
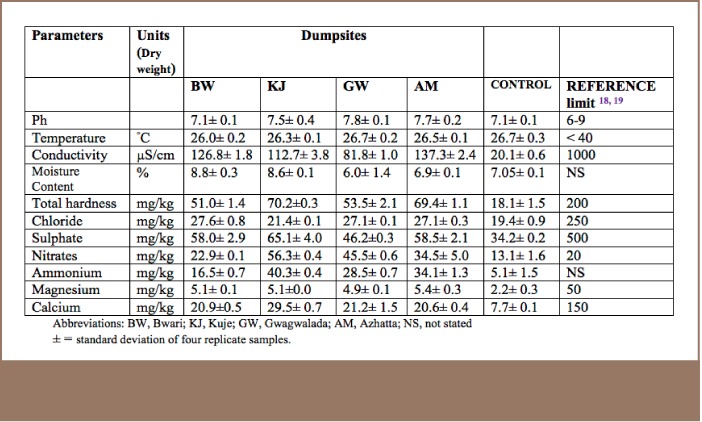
Physico-chemical Parameters of Soil Samples Collected from Dumpsites

The mean concentration of nitrates from the sample sites was higher than the reference site and WHO standards, as shown in Table 1. Due to the high solubility of nitrate in soil and its inability to retain anions, nitrates leach during rains and contaminate surface and ground waters.^20^ Except for zinc, the average concentrations for the other studied heavy metals were higher at all the dumpsites than the permissible levels stipulated by the reference^18^,^19^ (*Table 2*), even at the reference site.

**Table 2:**
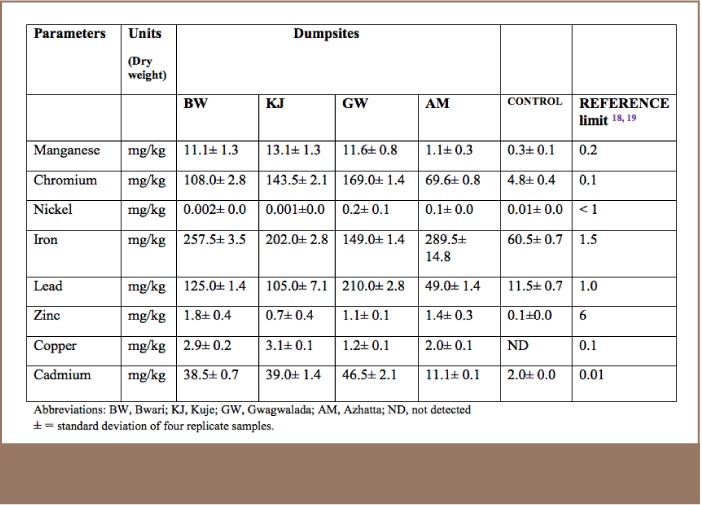
Heavy Metals Analysis of Soil Samples Collected from Dumpsites

Four genera of bacteria were identified: total heterotrophic bacteria, total coliform, Escherichia coli and Shigella/Salmonella. The results in Table 3 illustrate the high levels of total heterotrophic bacteria of the soil samples with regard to total coliform count, Escherichia coli, and Shigella/Salmonella, which were higher than the reference limits.^18^,^19^

**Table 3:**
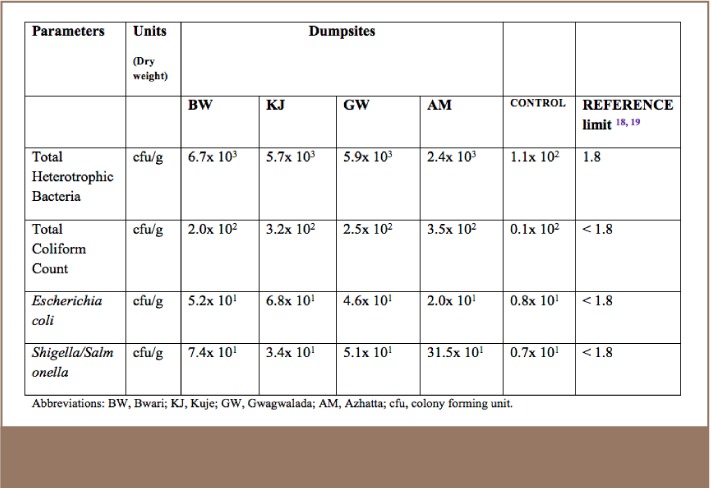
Microbial Analysis of Soil Samples Collected from Dumpsites

### Water

The mean temperature values of the water samples were within the range of the NESREA limit of 30°C. The mean concentrations for total suspended solids were higher in the Bwari and Kuje dumpsites, but not at the Gwagwalada and Azhatta dumpsites or the reference sample, which were within the standard limit as shown in Table 4. The mean concentration of total dissolved solids was lower than the WHO standard of 500 mg/l for the discharge of wastewater into surface water (*Table 4*).

**Table 4:**
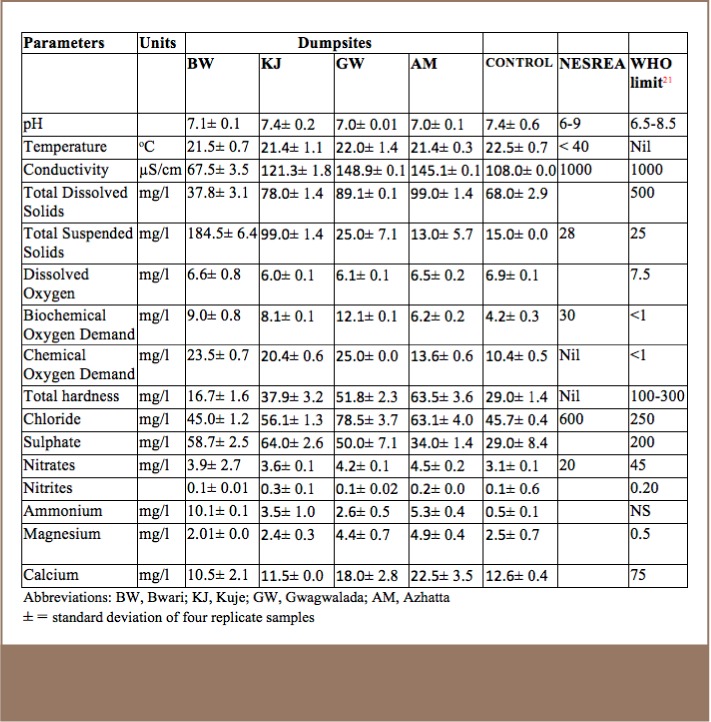
Physico-chemical Parameters of Water Samples Collected from the Groundwater near the Dumpsites

The mean conductivity values of the effluent from the dumpsites were very low compared to the NESREA and WHO guideline limits of 1000 μScm-3 for the effluent.

The mean biochemical oxygen demand values of the water samples were significantly higher than the WHO limits, but within the NESREA limits for effluent (*Table 4*). The mean concentrations of nitrate in the water samples were lower than the WHO limit of 45 mg/l.^21^ Iron concentrations in the Bwari dumpsite (1.7 mg/L) and Kuje dumpsite (2.1 mg/L) were above the standard limits, as shown in Table 5. The findings were similar to the values reported by Adeolu in the analysis of heavy metals in the effluent from an acid battery recycling plant.^22^

**Table 5:**
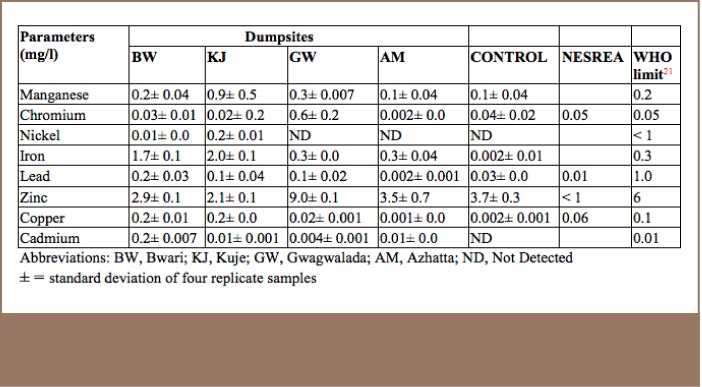
Heavy Metals Analysis of Water Samples Collected from the Groundwater near Dumpsites

Mean concentrations of lead were higher in the Bwari, Kuje and Gwagwalada dumpsites, but not at the Azhatta dumpsite, which had a low appreciable concentration (0.002 mg/l) below the NESREA and WHO guideline limits. In addition, the concentration of cadmium in the Bwari dumpsite was higher than the WHO limits. Cadmium is carcinogenic, and long term exposure contributes to kidney disease, lung damage and fragile bones.^23^

As shown in Table 6, the values of total heterotrophic bacteria, total coliform count, Escherichia coli and Shigella/Salmonella in the water samples collected from the wells near the selected dumpsites were higher than at the reference samples from the control site and WHO limits.

**Table 6:**
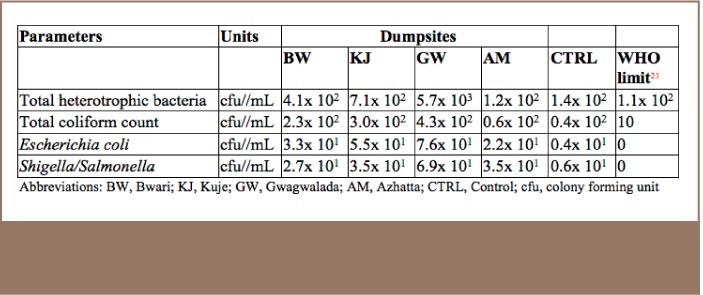
Microbial Burden of Water Samples Collected from Groundwater near Dumpsites

## Discussion

### Soil

Soil conductivity is primarily due to an increase in the concentration of soluble salts in the soil. Some metals like zinc and copper are needed for metabolism in organisms and concern over these metals lies in the thin line between their toxicity and essentiality.^22^ Under prolonged waterlogged conditions, soluble manganese leaches out of soil.^20^ Exposure to chromium can be carcinogenic, and chromium therefore poses a threat to public health.^24^ It is worth mentioning that metal ion concentrations in the selected dumpsites were higher than the thresholds set by the WHO even at the control site. Excess of some heavy metals in the environment can cause serious environmental health consequences because of their biomagnification and bioaccumulation.^6^

It should be noted that bacterial isolates with high frequency of occurrence are important human pathogens associated with a variety of infectious diseases such as gastroenteritis, typhoid fever, dysentery, cholera, and urinary tract infection.^25^ The source of these pathogens is attributed to the biological degradation of waste materials in the dumpsites over time.

### Water

Temperature has an important effect on other properties of wastewater. High conductivity values (high salt concentrations) increase the salinity of the receiving river, which may result in adverse ecological effects on the aquatic biota and therefore high salt concentrations are a potential health hazard.^26^,^27^ Biochemical oxygen demand reflects the presence of organic matter which will be oxidized biologically in the receiving water.^28^ The greater the decomposable matter present, the greater the oxygen demand and the greater the biochemical oxygen demand value. Continuous discharge of effluent with a high biochemical oxygen demand into a river has negative effects on the quality of fresh water and can cause harm to aquatic life, especially fish downstream, and is hazardous to the survival of the aquatic biota in the receiving stream.^22^ The concentrations of magnesium were very high, which could result in water hardness and pose a threat to public health.^5^

The high levels of nitrate can give rise to blue-eye syndrome in infants and pregnant women and when combined with phosphate causes eutrophication.^29^,^30^ Excess iron concentration in water impacts taste, imparts a reddish color, and increases the turbidity of water which promotes the growth of ferrous bacteria that accelerate the rusting process of metals that come in contact with water. The accumulation of metals in an aquatic environment has direct consequences to humans and the ecosystem. It should be noted that the ingestion of chromium in low concentrations is carcinogenic. When inhaled, chromium (hexavalent) damages the lining of the nose and throat, and irritates the lungs as well as the gastrointestinal tract.^22^ When swallowed, it upsets the stomach and damages the liver and kidneys. Some individuals have an allergic skin reaction after touching materials containing chromium.

Exposure to high concentrations of lead through drinking water or residing near a lead-contaminated toxic dump can cause brain dysfunction in children, neuro-behavioral changes in adults, hypertension and chronic kidney diseases.^22^,^31^ The mean levels of copper from the water samples were higher than the standard permissible limits, which can result in an astringent taste, discoloration, corrosion of pipes, fittings and utensils, and induce brain damage in mammals.^17^ In addition, the concentration of cadmium in the Bwari dumpsite was higher than the WHO limit. Cadmium is carcinogenic, and long term exposure can contribute to kidney disease, lung damage and fragile bones.^22^

The high incidence of fecal coliforms in these selected dumpsites was indicative of increasing pollution of groundwater by organic contaminants. Fecal coliforms have serious health implications and can cause urinary tract infections, bacteremia, meningitis, diarrhea and acute renal failure.^32^,^33^

## Conclusions

The findings on the impact of dumpsites on soil and ground water quality in satellite towns of the Federal Capital Territory revealed that the measured soil and water parameters exceeded the standard limits and pose significant health and environmental risks to nearby residents or users if care is not taken. There is a need for a standard policy framework that will guide the management and maintenance of dumpsites in these satellite towns either by the government or through a public-private partnership. There is also a need for greater public awareness by residents living near these dumpsites or those who use the wells or nearby stream water for domestic activities on the need to carry out adequate water treatment prior to use to prevent health problems. Proper methods of waste disposal need to be undertaken to ensure that the surrounding environment is not affected and to mitigate health hazards to the local population. At the household level, education on proper segregation of waste is needed and it should be ensured that all organic matter is sorted for biogas and compost, which is the preferred method for the sanitary disposal of waste.
